# Sex differences in survival outcomes of early-onset colorectal cancer

**DOI:** 10.1038/s41598-024-71999-8

**Published:** 2024-09-26

**Authors:** Abdelrahman Yousry Afify, Mohamed Hady Ashry, Hamsa Hassan

**Affiliations:** 1 School of Medicine, New Giza University (NGU), Giza, Egypt; 2 Internal Medicine Department, Cairo University Hospitals, Cairo, Egypt; 3 Medical Research Platform, Giza, Egypt; 4 Egypt-Japan University of Science and Technology (EJUST), New Borg El Arab, Alexandria Egypt; 5 Faculty of Pharmacy, Mansoura University, Mansoura, Egypt

**Keywords:** Survival, Cancer, Early-onset colorectal cancer, Sex disparities, Prognosis, Surveillance, Epidemiology, End results, Cancer epidemiology, Cancer, Gastrointestinal cancer, Colorectal cancer

## Abstract

Colorectal cancer (CRC) is one of the most fatal cancers in the United States. Although the overall incidence and mortality rates are declining, an alarming rise in early-onset colorectal cancer (EOCRC), defined as CRC diagnosis in patients aged < 50 years, was previously reported. Our study focuses on analyzing sex-specific differences in survival among EOCRC patients and comparing sex-specific predictors of survival in both males and females in the United States. We retrieved and utilized data from the Surveillance, Epidemiology, and End Results (SEER) program. EOCRC patients, between the ages of 20 and 49, were exclusively included. We conducted thorough survival analyses using Kaplan–Meier curves, log-rank tests, Cox regression models, and propensity score matching to control for potential biases. Our study included 58,667 EOCRC patients (27,662 females, 31,005 males) diagnosed between 2000 and 2017. The baseline characteristics at the time of diagnosis were significantly heterogeneous between males and females. Males exhibited significantly worse overall survival (OS), cancer-specific survival (CSS), and noncancer-specific survival (NCSS) in comparison to females in both the general cohort, and the matched cohort. Predictors of survival outcomes generally followed a similar pattern in both sexes except for minor differences. In conclusion, we identified sex as an independent prognostic factor of EOCRC, suggesting disparities in survival between sexes. Further understanding of the epidemiological and genetic bases of these differences could facilitate targeted, personalized therapeutic approaches for EOCRC.

## Introduction

Colorectal cancer (CRC) is the second most fatal cancer in the United States, with more than 50,000 reported deaths in 2024, following lung and bronchus cancer. An estimated 150,000 new cases have been diagnosed correspondingly, with a slight shift towards males compared to females^1^. Increased colorectal cancer screening and improved treatment strategies in the United States have contributed to a decline in overall incidence and mortality rates; however, a worrisome increase in the incidence of early-onset colorectal cancer (EOCRC), defined as CRC diagnosis in patients aged < 50 years, has been observed despite the declining of the overall incidence of CRC^2–4^. EOCRC exhibits more restricted pathological features and symptoms in comparison to later-onset colorectal cancer (LOCRC). Accordingly, patients are less likely to seek immediate medical care^5^. To our knowledge, the current upward trend has not been fully accounted for by various driving environmental and genetic risk factors with limited understanding of the precise epidemiology of EOCRC. However, the current belief is that the epidemiological profile of EOCRC patients is distinct from that of LOCRC patients^6^. Owing to the rising incidence of EOCRC, a surge in research investigating the clinical outcomes and prognostic factors of EOCRC was recently observed. Previous studies have suggested potential sex-based differences in survival outcomes among CRC patients^7^. However, the findings of these studies were often conflicting possibly due to their loose inclusion criteria that hindered drawing specific conclusions regarding this distinct population of CRC patients. Our study aims to investigate sex-specific differences in survival among EOCRC patients, and separately compare sex-specific predictors of survival in both males and females in the United States using population-level data.

## Methods

### Study design

The Strengthening the Reporting of Observational Studies in Epidemiology (STROBE) standards were followed in conducting our retrospective, observational cohort study^8^.

### Data source

This investigation was carried out using the Surveillance, Epidemiology, and End Results (SEER) program^9^. We retrieved the data of cancer patients from the SEER 17 registries dataset, which covers roughly 26.5% of the US population using the SEER*Stat software (version 8.4.3; https://seer.cancer.gov/seerstat/)^10^. Because this study uses anonymized SEER data, permission from an institutional review board was not required.

### Population selection

**Fig. 1:**
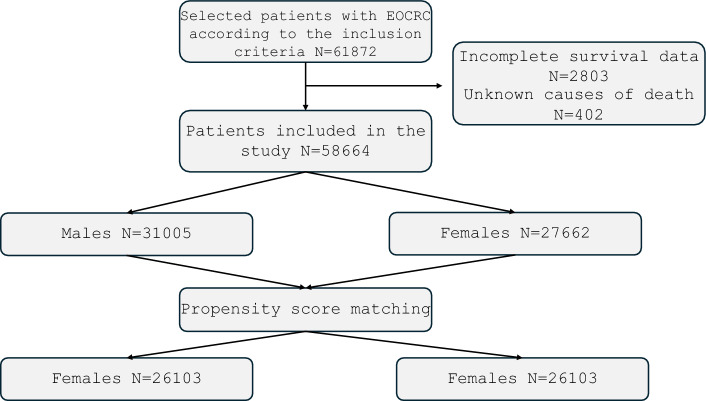
Flowchart of the patients screening process.

Patients’ eligibility was determined based on the following inclusion criteria: (1) Patients diagnosed with colorectal cancer (identified using the “site and morphology” recode based on the ICD-0–3/WHO 2008 definitions) (2) patients with microscopically confirmed malignant CRC (3) patients with a known age at diagnosis between the age of 20 and 49 (4) patients diagnosed between 2000 and 2017 (available SEER staging data). The exclusion criteria were as follows: (1) patients with incomplete survival times (2) patients with an unknown cause of death (3) patients identified through death certificate or autopsy only. The flowchart illustrating the selection of EOCRC patients is depicted in Fig. 1.

### Study variable

The main examined variable in this study was the patients’ sex classified by the SEER database as either male or female. Additionally, we collected the following variables for each patient included in our analysis: age at diagnosis, race, marital status at diagnosis, median household income, residential area, primary site of cancer, tumor histology (according to the SEER histology broad groupings variable groupings into adenocarcinomas (codes 8140–8389), cystic and mucinous tumors (codes 8440–8499), and the rest of tumor histology collectively as “Others”), and grade, cancer stage according to the SEER stage summary recode, and types of treatment modalities received. To avoid possible selection bias, all patients diagnosed with EOCRC who fit the inclusion criteria were included in the study regardless of the presence of missing baseline variables.

### Study outcomes

The outcomes of interest for this study were Overall survival (OS) defined as the duration between the time of CRC diagnosis and death of any cause or the date of last follow-up, cancer-specific survival (CSS) defined as the duration between the time of CRC diagnosis, and death attributed to the index CRC, and noncancer-specific survival (NCSS) defined as the duration between the time of CRC diagnosis and death attributed to other causes than the patients’ index cancer. We determined the causes of death using the SEER’s provided vital status recode, cause-specific, and other cause-of-death classifications.

### Statistical analysis

Categorical variables of males and females were described as frequencies and percentages, then compared using Pearson’s chi-squared test. We used Kaplan–Meier survival curves to investigate the crude survival of different patient cohorts. Differences in survival were compared using the log-rank test. Furthermore, univariable and multivariable survival analyses using Cox proportional hazards models were used to calculate the hazard ratios (HRs) and their respective 95% confidence intervals (CI), characterizing the impact of different variables on survival outcomes. Additionally, we used propensity score matching (PSM) to match male and female patients using the different baseline variables, eliminating possible biases and enhancing the robustness of the study’s results. The matched cohort was then examined using the same survival analysis pipeline. Survival analyses were carried out using Jamovi Software (version 2.3.28; https://www.jamovi.org/) while PSM analysis was conducted using the R software (version 3.6.3; https://cran.r-project.org/). All analyses were two-tailed and a statistical level of 0.05 was considered significant.

## Results

### Baseline characteristics

We retrieved and analyzed the data of 58,667 patients diagnosed with EOCRC from the SEER dataset. Out of the total cohort, 27,662 were females and 31,005 were males. We observed significant differences in the distribution of the two groups across all of the included baseline characteristics. Notably, males were diagnosed at an older age compared to females, and significant differences in race between the two groups were also observed. Males were more likely to be diagnosed with rectal cancer (42.2% vs 36.7%) while females had a higher percentage of left-sided colon disease compared to males (31.1% vs 26.6%). Additionally, differences in cancer stages were also observed with more males diagnosed with regional disease (40% vs 37.2%) and more females diagnosed with localized disease (34.8% vs 32%). Males were more likely to receive radiotherapy and chemotherapy, and less likely to have surgery compared to their female counterparts. Table 1 summarizes the baseline characteristics of patients with EOCRC.

**Table 1:** Baseline characteristics of patient diagnosed with early-onset colorectal cancer (EOCRC) from the SEER database.

	Female (N = 27,662)	Male (N = 31,005)	p-value
Age			< 0.001
20–29	1660.0 (6.0%)	1679.0 (5.4%)	
30–39	6153.0 (22.2%)	6500.0 (21.0%)	
40–49	19,849.0 (71.8%)	22,826.0 (73.6%)	
Race			< 0.001
White	20,083.0 (72.6%)	23,308.0 (75.2%)	
Black	4248.0 (15.4%)	4106.0 (13.2%)	
Asian or Pacific Islander	2717.0 (9.8%)	2951.0 (9.5%)	
American Indian/Alaska Native	330.0 (1.2%)	319.0 (1.0%)	
Unspecified	284.0 (1.0%)	321.0 (1.0%)	
Marital status			< 0.001^1^
Married	15,737.0 (56.9%)	17,470.0 (56.3%)	
Single	6918.0 (25.0%)	9000.0 (29.0%)	
Unmarried	3356.0 (12.1%)	2732.0 (8.8%)	
Unspecified	1651.0 (6.0%)	1803.0 (5.8%)	
Income			0.009
< $35 k	313.0 (1.1%)	366.0 (1.2%)	
$35 k—$50 k	2730.0 (9.9%)	2918.0 (9.4%)	
$50 k—$75 k	13,773.0 (49.8%)	15,171.0 (48.9%)	
> $75 k	10,846.0 (39.2%)	12,550.0 (40.5%)	
Area of residence			0.010
Metropolitan	24,391.0 (88.2%)	27,382.0 (88.3%)	
Nonmetropolitan	3168.0 (11.5%)	3550.0 (11.4%)	
Unspecified	103.0 (0.4%)	73.0 (0.2%)	
Primary site			< 0.001
Right-sided colon	7037.0 (25.4%)	7439.0 (24.0%)	
Transverse colon	1250.0 (4.5%)	1473.0 (4.8%)	
left-sided colon	8605.0 (31.1%)	8261.0 (26.6%)	
Rectum/Rectosigmoid junction	10,161.0 (36.7%)	13,090.0 (42.2%)	
Unspecified/NOS	609.0 (2.2%)	742.0 (2.4%)	
Grade			< 0.001
Grade I	3362.0 (12.2%)	3110.0 (10.0%)	
Grade II	15,490.0 (56.0%)	17,394.0 (56.1%)	
Grade III	3961.0 (14.3%)	4998.0 (16.1%)	
Grade IV	548.0 (2.0%)	616.0 (2.0%)	
Unspecified	4301.0 (15.5%)	4887.0 (15.8%)	
Histology			< 0.001
Adenocarcinomas	24,335.0 (88.0%)	26,805.0 (86.5%)	
Cystic/mucinous	2696.0 (9.7%)	3555.0 (11.5%)	
Others	631.0 (2.3%)	645.0 (2.1%)	
Stage			< 0.001
Localized	9620.0 (34.8%)	9924.0 (32.0%)	
Regional	10,290.0 (37.2%)	12,396.0 (40.0%)	
Distant	7045.0 (25.5%)	7785.0 (25.1%)	
Unspecified	707.0 (2.6%)	900.0 (2.9%)	
Radiotherapy			< 0.001
No/Unknown	22,478.0 (81.3%)	23,481.0 (75.7%)	
Yes	5184.0 (18.7%)	7524.0 (24.3%)	
Chemotherapy			< 0.001
No/Unknown	12,213.0 (44.2%)	12,933.0 (41.7%)	
Yes	15,449.0 (55.8%)	18,072.0 (58.3%)	
Surgery			< 0.001
No/Unknown	3339.0 (12.1%)	4679.0 (15.1%)	
Yes	24,323.0 (87.9%)	26,326.0 (84.9%)	

### Differences in survival between males and females

**Fig. 2:**
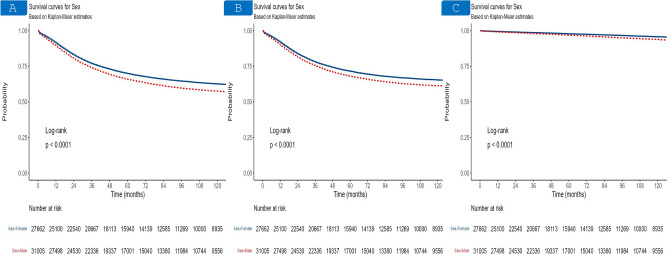
Kaplan–Meier curves of the general SEER cohort. Overall survival (**A**), cancer-specific survival (**B**), and noncancer-specific survival (**C**) in female, and male early-onset colorectal cancer patients.

The 1-year, 3-year, and 5-year OS probabilities for males were 89.1%,73.8%, and 65.7%, respectively. While The 1-year, 3-year, and 5-year OS probability for females were 91.3%, 76.9%, and 69.9%, respectively. On the other hand, the 1-year, 3-year, and 5-year CSS probability were 89.9%,75.3%, and 67.9% for males, and 91.9%,78%, and 71.5% for females. In terms of NCSS, the 1-year, 3-year, and 5-year survival for males were 99.14%,98%, and 96.79%, and 99.41%, 98.59%, and 97.78% for females, respectively. Kaplan–Meier survival estimates, and log-rank tests demonstrated worse survival of males compared to females in regards to OS, CS, and NCSS (P < 0.0001) (Fig. 2). In the multivariable Cox regression model, male sex was associated with worse OS (HR = 1.19, 95% CI 1.16–1.22, P < 0.001), CSS (HR = 1.15, 95% CI 1.11–1.28, P < 0.001), and NCSS (HR = 1.56, 95% CI 1.44–1.69, P < 0.001) (Table 2 and Supplementary Table 1).

**Table 2:** Cox regression models for sex in the general cohort and the post-propensity score matching cohort.

	OS				CSS				NCSS			
	Univariable HR (95% CI)	p-value	Multivariable HR (95% CI)	p-value	Univariable HR (95% CI)	p-value	Multivariable HR (95% CI)	p-value	Univariable HR (95% CI)	P-value	Multivariable HR (95% CI)	p-value
General cohort												
Female	Ref	–	Ref	–	Ref	–	Ref	–	Ref	–	Ref	–
Male	1.19 (1.16–1.22)	< 0.001	1.19 (1.16–1.22)	< 0.001	1.15 (1.12–1.19)	< 0.001	1.15 (1.11–1.18)	< 0.001	1.52 (1.41–1.65)	< 0.001	1.56 (1.44–1.69)	< 0.001
Post-PSM												
Female	Ref	–	Ref	–	Ref	–	Ref	–	Ref	–	Ref	–
Male	1.12 (1.09–1.16)	< 0.001	1.18 (1.15–1.21)	< 0.001	1.08 (1.05–1.11)	< 0.001	1.14 (1.10–1.17)	< 0.001	1.51 (1.39–1.64)	< 0.001	1.58 (1.45–1.71)	< 0.001

Multivariable analysis was adjusted for all the baseline variables including patients’ age groups, race, marital status, income, residence, tumors’ site, grade, histology, stage, and treatment modalities received.

*OS* Overall survival, *CSS* Cancer-Specific Survival, *NCSS* Noncancer-Specific Survival, *HR* Hazard Ratio, *CI* Confidence Interval, *PSM* Propensity Score Matching, *Ref* Reference.

**Fig. 3:**
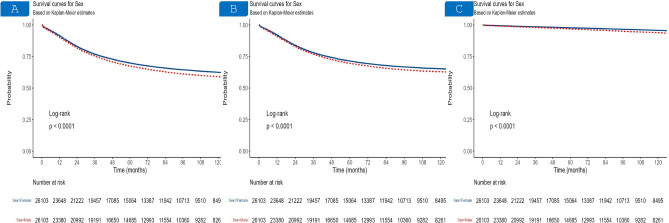
Kaplan–Meier curves of the post-PSM cohort. Overall survival (**A**), cancer-specific survival (**B**), and noncancer-specific survival (**C**) in female, and male early-onset colorectal cancer patients.

Using PSM, we matched males and females in a 1:1 ratio to control for the possible biases introduced by the study’s retrospective design. The PSM-matched cohort included 26,103 patient pairs without demonstrating significant differences between the two groups (Supplementary Table 2). The survival of the matched cohort closely mirrored the general cohort with males showing worse outcomes (Fig. 3). Compared to females, male sex continued to be associated with worse OS (HR = 1.18, 95% CI 1.15–1.21, P < 0.001), CSS (HR = 1.14, 95% CI 1.1–1.17, P < 0.001), and NCSS (HR = 1.58, 95% CI 1.45–1.71, P < 0.001) (Table 2 and Supplementary Table 3). These results confirm the previous conclusions of the general cohort.

We conducted a subgroup analysis of all baseline variables stratifications comparing the survival of males and females with subsequent adjustment using multivariable models. Regarding OS, males had a statistically significant worse survival compared to females in all subgroups except for the American Indian/Alaska Native subgroup (P = 0.43), the < $35k median household incomesubgroup (P = 0.779), and the transverse colon subgroup (P = 0.67). Males also had a statistically significant worse CSS compared to females in all subgroups except for the Asian or Pacific Islander subgroup (P = 0.138), the American Indian/Alaska Native subgroup (P = 0.714), the < $35k median household income subgroup (P = 0.92), the transverse colon subgroup (P = 0.682), and the grade IV tumor subgroup (P = 0.056). Additionally, male sex was associated with worse NCSS in all subgroups except for the 20–29 age subgroup (P = 0.863), the American Indian/Alaska Native subgroup (P = 0.219), the < $35k median household income subgroup (P = 0.409), the transverse colon subgroup(P = 0.054), the Cystic/mucinous histology subgroup (P = 0.106), and the grades III and IV tumors subgroups (P = 0.087 and P = 0.197, respectively) (Supplementary Table 4).

### Predictors of survival in males and females

The statistically significant factors affecting OS of male EOCRC in multivariable analysis were older age at diagnosis (40–49 years old vs 20–29 years old, HR = 1.18, P < 0.001), black race (vs white, HR = 1.26, P < 0.001), single and unmarried status (vs married, HR = 1.34, P < 0.001, and HR = 1.36, P < 0.001, respectively), a higher median income (> $75k vs < $35k, HR = 0.76, P = 0.001), tumor grades II, III, and IV (vs grade I, HR = 1.4, P < 0.001, HR = 2.18, P < 0.001, and HR = 2.5, P < 0.001, respectively), Cystic/mucinous histology (vs adenocarcinoma, HR = 1.24, P < 0.001), regional, and distant stages (vs localized stage, HR = 2.42, P < 0.001, and HR = 10.98, P < 0.001, respectively), and receiving radiotherapy, chemotherapy, and surgery (vs No/unknown status, HR = 1.09, P < 0.001, HR = 0.9, P < 0.001, and HR = 0.4, P < 0.001, respectively) (Table 3).

Factor significantly affecting CSS in males generally followed the same pattern for predictors of OS except for age which was not associated with CSS (40–49 years old vs 20–29 years old, HR = 1.05, P = 0.253). Factors affecting NCSS were also comparable to OS variables except for some distinctions. A higher median income, regional stage, and tumor histology did not affect NCSS (> $75k vs < $35k, P = 0.119, regional vs localized, P = 0.123, and Cystic/mucinous vs adenocarcinoma, P = 0.783, respectively). Moreover, rectal cancers and only tumor grade IV were associated with NCSS (vs right-sided colon cancer, HR = 0.78, P = 0.002, and vs grade I, HR = 1.65, P = 0.017, respectively) (Table 3).

**Table 3:** Cox regression models for survival in male early-onset colorectal cancer (EOCRC) patients.

	OS				CSS				NCSS			
	Univariable HR (95% CI)	P-value	Multivariable HR (95% CI)	P-value	Univariable HR (95% CI)	P-value	Multivariable HR (95% CI)	P-value	Univariable HR (95% CI)	P-value	Multivariable HR (95% CI)	P-value
Age												
20–29	Ref	–	Ref	–	Ref	–	Ref	–	Ref	–	Ref	–
30–39	1.08 (0.99–1.18)	0.09	1.06 (0.96–1.16)	0.242	1.06 (0.96–1.16)	0.249	1.00 (0.91–1.10)	0.948	1.51 (1.06–2.15)	0.024	1.81 (1.26–2.59)	0.001
40–49	1.19 (1.09–1.29)	< 0.001	1.18 (1.08–1.28)	< 0.001	1.09 (1.00–1.19)	0.042	1.05 (0.96–1.15)	0.253	2.60 (1.86–3.63)	< 0.001	3.19 (2.27–4.47)	< 0.001
Race												
White	Ref	–	Ref	–	Ref	–	Ref	–	Ref	–	Ref	–
Black	1.39 (1.33–1.46)	< 0.001	1.26 (1.20–1.32)	< 0.001	1.37 (1.31–1.45)	< 0.001	1.26 (1.20–1.33)	< 0.001	1.52 (1.33–1.72)	< 0.001	1.25 (1.10–1.43)	0.001
Asian or Pacific Islander	0.93 (0.88–0.99)	0.032	1.01 (0.95–1.07)	0.786	0.97 (0.91–1.04)	0.412	1.04 (0.97–1.11)	0.247	0.68 (0.55–0.83)	< 0.001	0.78 (0.64–0.96)	0.018
American Indian/Alaska Native	1.23 (1.05–1.44)	0.011	0.86 (0.72–1.03)	0.097	1.16 (0.97–1.38)	0.096	0.82 (0.68–1.00)	0.052	1.73 (1.17–2.56)	0.006	1.39 (0.88–2.20)	0.153
Unspecified	0.10 (0.06–0.16)	< 0.001	0.19 (0.11–0.32)	< 0.001	0.11 (0.07–0.19)	< 0.001	0.25 (0.15–0.42)	< 0.001	0.00 (0.00-Inf)	0.972	0.00 (0.00-Inf)	0.973
Marital status												
Married	Ref	–	Ref	–	Ref	–	Ref	–	Ref	–	Ref	–
Single	1.51 (1.45–1.57)	< 0.001	1.34 (1.29–1.39)	< 0.001	1.46 (1.40–1.52)	< 0.001	1.27 (1.22–1.32)	< 0.001	1.93 (1.73–2.15)	< 0.001	2.00 (1.79–2.24)	< 0.001
Unmarried	1.56 (1.47–1.65)	< 0.001	1.36 (1.29–1.44)	< 0.001	1.45 (1.36–1.54)	< 0.001	1.26 (1.18–1.34)	< 0.001	2.49 (2.15–2.88)	< 0.001	2.26 (1.95–2.62)	< 0.001
Unspecified	0.87 (0.79–0.94)	0.001	0.98 (0.90–1.07)	0.616	0.82 (0.75–0.90)	< 0.001	0.96 (0.87–1.06)	0.397	1.19 (0.96–1.49)	0.119	1.16 (0.92–1.45)	0.208
Income												
< $35 k	Ref	–	Ref	–	Ref	–	Ref	–	Ref	–	Ref	–
$35 k—$50 k	1.05 (0.89–1.23)	0.549	1.00 (0.85–1.18)	0.983	1.04 (0.87–1.23)	0.669	0.99 (0.83–1.18)	0.905	1.13 (0.72–1.75)	0.599	1.07 (0.68–1.66)	0.773
$50 k—$75 k	0.90 (0.77–1.04)	0.16	0.94 (0.80–1.11)	0.492	0.91 (0.77–1.07)	0.254	0.95 (0.79–1.13)	0.531	0.81 (0.53–1.24)	0.329	0.89 (0.57–1.40)	0.626
> $75 k	0.73 (0.63–0.86)	< 0.001	0.79 (0.67–0.93)	0.005	0.76 (0.64–0.89)	0.001	0.80 (0.67–0.95)	0.013	0.58 (0.38–0.89)	0.013	0.70 (0.44–1.10)	0.119
Area of residence												
Metropolitan	Ref	–	Ref	–	Ref	–	Ref	–	Ref	–	Ref	–
Nonmetropolitan	1.17 (1.11–1.23)	< 0.001	1.05 (0.99–1.12)	0.111	1.13 (1.07–1.19)	< 0.001	1.04 (0.97–1.12)	0.22	1.43 (1.25–1.63)	< 0.001	1.10 (0.93–1.31)	0.261
Unspecified	0.98 (0.69–1.41)	0.922	1.73 (1.16–2.59)	0.007	0.86 (0.57–1.29)	0.463	1.61 (1.02–2.54)	0.04	1.87 (0.89–3.94)	0.098	1.74 (0.72–4.17)	0.215
Primary site												
Right-sided colon	Ref	–	Ref	–	Ref	–	Ref	–	Ref	–	Ref	–
Transverse colon	1.03 (0.94–1.12)	0.577	1.00 (0.91–1.09)	0.924	1.01 (0.92–1.11)	0.832	0.97 (0.88–1.07)	0.572	1.11 (0.89–1.39)	0.354	1.12 (0.89–1.40)	0.328
left-sided colon	1.01 (0.96–1.06)	0.779	0.99 (0.94–1.04)	0.698	1.02 (0.96–1.07)	0.555	0.99 (0.94–1.04)	0.691	0.95 (0.83–1.09)	0.464	0.93 (0.81–1.07)	0.31
Rectum/Rectosigmoid junction	1.01 (0.96–1.05)	0.782	0.95 (0.90–1.01)	0.085	1.03 (0.98–1.08)	0.234	0.97 (0.91–1.03)	0.304	0.86 (0.76–0.98)	0.019	0.78 (0.67–0.91)	0.002
Unspecified	2.31 (2.10–2.54)	< 0.001	1.50 (1.36–1.66)	< 0.001	2.37 (2.14–2.63)	< 0.001	1.51 (1.35–1.67)	< 0.001	1.87 (1.41–2.48)	< 0.001	1.49 (1.10–2.01)	0.01
Grade												
Grade I	Ref	–	Ref	–	Ref	–	Ref	–	Ref	–	Ref	–
Grade II	1.85 (1.71–2.00)	< 0.001	1.40 (1.29–1.52)	< 0.001	2.04 (1.87–2.23)	< 0.001	1.45 (1.33–1.59)	< 0.001	1.17 (0.98–1.38)	0.081	1.14 (0.96–1.36)	0.138
Grade III	3.43 (3.16–3.73)	< 0.001	2.18 (2.00–2.37)	< 0.001	4.06 (3.71–4.46)	< 0.001	2.38 (2.17–2.62)	< 0.001	1.15 (0.94–1.42)	0.183	1.13 (0.91–1.40)	0.252
Grade IV	3.85 (3.39–4.38)	< 0.001	2.50 (2.19–2.85)	< 0.001	4.39 (3.83–5.04)	< 0.001	2.66 (2.31–3.06)	< 0.001	1.78 (1.19–2.67)	0.005	1.65 (1.09–2.47)	0.017
Unspecified	2.14 (1.96–2.33)	< 0.001	1.39 (1.28–1.52)	< 0.001	2.37 (2.15–2.61)	< 0.001	1.43 (1.30–1.58)	< 0.001	1.31 (1.08–1.59)	0.007	1.20 (0.99–1.47)	0.069
Histology												
Adenocarcinomas	Ref	–	Ref	–	Ref	–	Ref	–	Ref	–	Ref	–
Cystic/mucinous	1.53 (1.46–1.61)	< 0.001	1.24 (1.18–1.31)	< 0.001	1.61 (1.52–1.69)	< 0.001	1.27 (1.21–1.34)	< 0.001	1.04 (0.89–1.22)	0.639	1.02 (0.87–1.20)	0.783
Others	1.89 (1.70–2.09)	< 0.001	1.18 (1.06–1.31)	0.003	1.89 (1.70–2.12)	< 0.001	1.17 (1.05–1.32)	0.007	1.83 (1.38–2.44)	< 0.001	1.28 (0.95–1.73)	0.111
Stage												
Localized	Ref	–	Ref	–	Ref	–	Ref	–	Ref	–	Ref	–
Regional	2.56 (2.42–2.72)	< 0.001	2.42 (2.27–2.58)	< 0.001	3.73 (3.47–4.01)	< 0.001	3.42 (3.16–3.70)	< 0.001	0.99 (0.89–1.11)	0.913	1.10 (0.97–1.25)	0.123
Distant	14.71 (13.90–15.56)	< 0.001	10.98 (10.28–11.72)	< 0.001	23.18 (21.59–24.89)	< 0.001	16.58 (15.31–17.96)	< 0.001	1.70 (1.46–1.99)	< 0.001	1.75 (1.47–2.10)	< 0.001
Unspecified	3.09 (2.75–3.48)	< 0.001	1.74 (1.54–1.97)	< 0.001	4.25 (3.71–4.86)	< 0.001	2.34 (2.03–2.69)	< 0.001	1.56 (1.22–1.99)	< 0.001	1.19 (0.91–1.56)	0.207
Radiotherapy												
No/Unknown	Ref	–	Ref	–	Ref	–	Ref	–	Ref	–	Ref	–
Yes	1.17 (1.12–1.21)	< 0.001	1.09 (1.04–1.15)	< 0.001	1.19 (1.14–1.24)	< 0.001	1.08 (1.03–1.14)	0.003	0.99 (0.88–1.11)	0.861	1.34 (1.14–1.58)	< 0.001
Chemotherapy												
No/Unknown	Ref	–	Ref	–	Ref	–	Ref	–	Ref	–	Ref	–
Yes	2.21 (2.13–2.30)	< 0.001	0.90 (0.86–0.94)	< 0.001	2.65 (2.53–2.76)	< 0.001	0.93 (0.89–0.98)	0.009	0.84 (0.76–0.93)	< 0.001	0.67 (0.59–0.77)	< 0.001
Surgery												
No/Unknown	Ref	–	Ref	–	Ref	–	Ref	–	Ref	–	Ref	–
Yes	0.23 (0.22–0.24)	< 0.001	0.40 (0.38–0.42)	< 0.001	0.21 (0.20–0.22)	< 0.001	0.38 (0.36–0.40)	< 0.001	0.57 (0.49–0.67)	< 0.001	0.70 (0.58–0.84)	< 0.001

*OS* overall survival, *CSS* cancer-specific survival, *NCSS* noncancer-specific survival, *HR* hazard ratio, *CI* confidence interval, *Ref *reference.

In females, the statistically significant factors affecting OS were older age at diagnosis (40–49 years old vs 20–29 years old, HR = 1.13, P < 0.001), black race, and Asian or pacific islander (vs white, HR = 1.32, P < 0.001, and HR = 1.09, P = 0.013 respectively), single and unmarried status (vs married, HR = 1.29, P < 0.001, and HR = 1.23, P < 0.001, respectively), a higher median income ($50k—$75k vs < $35k, HR = 0.82, P = 0.036, and > $75k vs < $35k, HR = 0.77, P = 0.006), residence (nonmetropolitan vs metropolitan, HR = 1.15, P < 0.001), patients with transverse colon, and rectal tumors (vs right-sided colon tumors, HR = 1.1, P = 0.041, and HR = 0.9, P = 0.001, respectively), tumor grades II, II, and IV (vs grade I, HR = 1.63, P < 0.001, HR = 2.59, P < 0.001, and HR = 2.72, P < 0.001, respectively), Cystic/mucinous histology (vs adenocarcinoma, HR = 1.15, P < 0.001), regional, and distant stages (vs localized stage, HR = 3.04, P < 0.001, and HR = 15.11, P < 0.001, respectively), and receiving radiotherapy, and surgery (vs No/unknown status, HR = 1.09, P < 0.001, and HR = 0.4, P < 0.001, respectively) (Table 4).

Factors significantly affecting CSS in females generally followed the same pattern for predictors of OS except for age at diagnosis, and median income which were not associated with CSS. Compared to the OS factors, NCSS was not related to area of residence, tumor grade (except for grade III), and radiotherapy. However, chemotherapy was associated with better NCSS (vs No/unknown status, HR = 0.62, P < 0.001) (Table 4).

**Table 4:** Cox regression models for survival in female early-onset colorectal cancer (EOCRC) patients.

	OS				CSS				NCSS			
	Univariable HR (95% CI)	P-value	Multivariable HR (95% CI)	P-value	Univariable HR (95% CI)	P-value	Multivariable HR (95% CI)	P-value	Univariable HR (95% CI)	P-value	Multivariable HR (95% CI)	P-value
Age												
20–29	Ref	–	Ref	–	Ref	–	Ref	–	Ref	–	Ref	–
30–39	1.15 (1.04–1.28)	0.006	0.97 (0.88–1.07)	0.567	1.15 (1.04–1.28)	0.009	0.94 (0.84–1.04)	0.232	1.20 (0.84–1.71)	0.309	1.38 (0.97–1.98)	0.075
40–49	1.28 (1.16–1.40)	< 0.001	1.13 (1.03–1.25)	0.012	1.25 (1.14–1.38)	< 0.001	1.08 (0.98–1.19)	0.141	1.53 (1.10–2.13)	0.011	1.82 (1.30–2.54)	< 0.001
Race												
White	Ref	–	Ref	–	Ref	–	Ref	–	Ref	–	Ref	–
Black	1.35 (1.28–1.42)	< 0.001	1.32 (1.25–1.39)	< 0.001	1.32 (1.25–1.39)	< 0.001	1.32 (1.25–1.40)	< 0.001	1.59 (1.37–1.86)	< 0.001	1.30 (1.11–1.53)	0.001
Asian or Pacific Islander	1.08 (1.01–1.15)	0.028	1.09 (1.02–1.17)	0.013	1.11 (1.04–1.19)	0.002	1.11 (1.03–1.19)	0.005	0.74 (0.57–0.95)	0.019	0.85 (0.66–1.10)	0.225
American Indian/Alaska Native	1.16 (0.97–1.38)	0.104	0.96 (0.78–1.19)	0.732	1.08 (0.89–1.31)	0.444	0.91 (0.73–1.15)	0.447	1.90 (1.22–2.96)	0.005	1.57 (0.90–2.72)	0.111
Unspecified	0.12 (0.07–0.21)	< 0.001	0.22 (0.12–0.39)	< 0.001	0.11 (0.06–0.20)	< 0.001	0.22 (0.12–0.42)	< 0.001	0.21 (0.05–0.86)	0.03	0.21 (0.05–0.87)	0.031
Marital Status												
Married	Ref	–	Ref	–	Ref	–	Ref	–	Ref	–	Ref	–
Single	1.34 (1.28–1.41)	< 0.001	1.29 (1.23–1.35)	< 0.001	1.30 (1.23–1.36)	< 0.001	1.24 (1.18–1.30)	< 0.001	1.88 (1.63–2.18)	< 0.001	1.83 (1.57–2.13)	< 0.001
Unmarried	1.32 (1.24–1.40)	< 0.001	1.23 (1.16–1.30)	< 0.001	1.25 (1.18–1.33)	< 0.001	1.17 (1.10–1.24)	< 0.001	2.04 (1.72–2.42)	< 0.001	1.88 (1.58–2.24)	< 0.001
Unspecified	0.72 (0.65–0.80)	< 0.001	0.90 (0.81–1.00)	0.042	0.68 (0.61–0.76)	< 0.001	0.89 (0.80–1.00)	0.045	1.11 (0.84–1.49)	0.458	0.98 (0.73–1.32)	0.898
Income												
< $35 k	Ref	–	Ref	–	Ref	–	Ref	–	Ref	–	Ref	–
$35 k—$50 k	0.91 (0.76–1.09)	0.302	0.89 (0.74–1.07)	0.202	0.97 (0.80–1.18)	0.763	0.96 (0.79–1.17)	0.661	0.63 (0.41–0.98)	0.039	0.59 (0.38–0.92)	0.019
$50 k—$75 k	0.79 (0.67–0.94)	0.007	0.82 (0.69–0.99)	0.036	0.87 (0.72–1.05)	0.141	0.90 (0.74–1.10)	0.318	0.43 (0.29–0.66)	< 0.001	0.45 (0.29–0.71)	0.001
> $75 k	0.70 (0.59–0.83)	< 0.001	0.77 (0.64–0.93)	0.006	0.77 (0.64–0.93)	0.007	0.85 (0.69–1.04)	0.121	0.36 (0.24–0.54)	< 0.001	0.39 (0.25–0.63)	< 0.001
Area of Residence												
Metropolitan	Ref	–	Ref	–	Ref	–	Ref	–	Ref	–	Ref	–
Nonmetropolitan	1.17 (1.10–1.24)	< 0.001	1.15 (1.07–1.24)	< 0.001	1.14 (1.07–1.21)	< 0.001	1.15 (1.06–1.25)	< 0.001	1.43 (1.20–1.69)	< 0.001	1.10 (0.87–1.38)	0.424
Unspecified	1.15 (0.84–1.56)	0.393	1.84 (1.26–2.69)	0.001	1.05 (0.74–1.47)	0.792	1.80 (1.19–2.72)	0.005	2.05 (0.97–4.31)	0.059	1.79 (0.71–4.53)	0.221
Primary Site												
Right-sided colon	Ref	–	Ref	–	Ref	–	Ref	–	Ref	–	Ref	–
Transverse colon	1.11 (1.01–1.22)	0.027	1.10 (1.00–1.22)	0.041	1.11 (1.00–1.22)	0.048	1.10 (0.99–1.22)	0.067	1.16 (0.87–1.54)	0.315	1.18 (0.89–1.57)	0.255
left-sided colon	0.94 (0.89–0.99)	0.024	0.98 (0.93–1.04)	0.527	0.96 (0.91–1.01)	0.121	0.98 (0.93–1.04)	0.602	0.81 (0.69–0.95)	0.012	0.90 (0.76–1.07)	0.241
Rectum/Rectosigmoid junction	0.80 (0.76–0.84)	< 0.001	0.90 (0.84–0.96)	0.001	0.80 (0.76–0.85)	< 0.001	0.90 (0.84–0.97)	0.003	0.78 (0.67–0.92)	0.003	0.79 (0.65–0.96)	0.017
Unspecified	2.19 (1.96–2.44)	< 0.001	1.24 (1.11–1.38)	< 0.001	2.27 (2.03–2.55)	< 0.001	1.25 (1.11–1.40)	< 0.001	1.42 (0.94–2.14)	0.092	1.12 (0.73–1.70)	0.611
Grade												
Grade I	Ref	–	Ref	–	Ref	–	Ref	–	Ref	–	Ref	–
Grade II	2.22 (2.03–2.42)	< 0.001	1.63 (1.49–1.79)	< 0.001	2.49 (2.26–2.75)	< 0.001	1.74 (1.57–1.92)	< 0.001	1.07 (0.87–1.32)	0.534	1.07 (0.86–1.33)	0.522
Grade III	4.56 (4.15–5.01)	< 0.001	2.59 (2.35–2.85)	< 0.001	5.30 (4.78–5.88)	< 0.001	2.80 (2.52–3.12)	< 0.001	1.49 (1.16–1.91)	0.002	1.41 (1.09–1.83)	0.01
Grade IV	4.46 (3.86–5.15)	< 0.001	2.72 (2.35–3.15)	< 0.001	5.14 (4.42–5.99)	< 0.001	2.94 (2.52–3.43)	< 0.001	1.56 (0.95–2.57)	0.082	1.42 (0.86–2.36)	0.172
Unspecified	2.39 (2.17–2.64)	< 0.001	1.56 (1.41–1.73)	< 0.001	2.63 (2.36–2.93)	< 0.001	1.63 (1.46–1.83)	< 0.001	1.37 (1.08–1.74)	0.01	1.20 (0.94–1.54)	0.143
Histology												
Adenocarcinomas	Ref	–	Ref	–	Ref	–	Ref	–	Ref	–	Ref	–
Cystic/mucinous	1.81 (1.71–1.91)	< 0.001	1.15 (1.09–1.23)	< 0.001	1.86 (1.75–1.97)	< 0.001	1.16 (1.09–1.23)	< 0.001	1.37 (1.12–1.67)	0.002	1.22 (0.99–1.50)	0.062
Others	1.62 (1.44–1.82)	< 0.001	1.06 (0.94–1.20)	0.325	1.54 (1.36–1.75)	< 0.001	1.02 (0.90–1.16)	0.757	2.25 (1.67–3.04)	< 0.001	1.56 (1.13–2.15)	0.007
Stage												
Localized	Ref	–	Ref	–	Ref	–	Ref	–	Ref	–	Ref	–
Regional	3.30 (3.07–3.55)	< 0.001	3.04 (2.80–3.29)	< 0.001	4.75 (4.34–5.20)	< 0.001	4.21 (3.82–4.64)	< 0.001	1.14 (0.99–1.31)	0.073	1.39 (1.18–1.65)	< 0.001
Distant	19.85 (18.51–21.28)	< 0.001	15.11 (13.95–16.37)	< 0.001	30.87 (28.29–33.68)	< 0.001	22.52 (20.44–24.81)	< 0.001	1.98 (1.65–2.38)	< 0.001	2.10 (1.69–2.61)	< 0.001
Unspecified	3.30 (2.83–3.84)	< 0.001	2.13 (1.82–2.49)	< 0.001	4.33 (3.63–5.16)	< 0.001	2.79 (2.33–3.34)	< 0.001	1.76 (1.28–2.41)	< 0.001	1.23 (0.87–1.73)	0.24
Radiotherapy												
No/Unknown	Ref	–	Ref	–	Ref	–	Ref	–	Ref	–	Ref	–
Yes	1.16 (1.11–1.22)	< 0.001	1.09 (1.02–1.15)	0.008	1.19 (1.13–1.25)	< 0.001	1.09 (1.02–1.16)	0.009	0.96 (0.82–1.13)	0.643	1.22 (0.98–1.51)	0.074
Chemotherapy												
No/Unknown	Ref	–	Ref	–	Ref	–	Ref	–	Ref	–	Ref	–
Yes	2.77 (2.65–2.90)	< 0.001	0.95 (0.90–1.00)	0.065	3.29 (3.13–3.46)	< 0.001	1.00 (0.94–1.06)	0.908	0.86 (0.76–0.97)	0.018	0.62 (0.52–0.73)	< 0.001
Surgery												
No/Unknown	Ref	–	Ref	–	Ref	–	Ref	–	Ref	–	Ref	–
Yes	0.24 (0.23–0.26)	< 0.001	0.40 (0.38–0.42)	< 0.001	0.23 (0.22–0.24)	< 0.001	0.40 (0.37–0.42)	< 0.001	0.45 (0.37–0.54)	< 0.001	0.50 (0.40–0.62)	< 0.001

*OS* overall survival, *CSS* cancer-specific survival, *NCSS* noncancer-specific survival, *HR* hazard ratio, *CI* confidence interval, Ref reference.

## Discussion

There is controversial evidence on sex differences in survival rates of CRC. Some studies suggested that females have superior survival rates than males^11–14^. However, others did not show significant survival differences^15,16^. Different studies assessed sex-related differences in prognosis and survival among older and later-onset CRC patients^14,17–19^, yet the current research focuses on investigating sex differences in survival among EOCRC and sex-specific predictors of survival in both sexes in the recent years in the United states. We found that men with EOCRC have worse survival outcomes than women. Kaplan–Meier survival and log-rank test showed worse survival of males when compared to females, in terms of OS, CSS, and NCSS (p < 0.0001). Additionally, multivariable Cox regression models confirmed the association between male sex and worse survival outcomes. Moreover, the analysis of matched cohorts showed similar results. These findings were consistent across most subgroups and cancer stages.

Majek et.al conducted a population-based analysis of 164,996 CRC patients in Germany. They found that age-adjusted 5-year survival was longer in females than in males 64.5% vs 61.9%, p < 0.0001). Notably, the survival advantage in women was highest in patients < 45 years old. In a multivariable analysis, women continued to show a survival advantage over men, even after adjusting for CRC stage and subsite, in patients < 56-year-old, but not in older patients^14^. Similarly, Yang et. al conducted a meta-analysis of studies reporting survival differences between male and female sexes among CRC patients. It showed that females had significantly longer OS (HR: 0.87; 95% CI 0.85–0,89) and CSS (HR: 0.92; 95% CI 0.89–0.95) than males^18^. Interestingly, our study revealed that male sex was adversely associated with OS, CSS, and NCSS. Therefore, we suggest that the bad prognosis is not only cancer-related, but may also be non-cancer-related. These findings are supported by the work of Samawi et al. who revealed a comparable conclusion in early-stage CRC patients concerning the OS. In a multivariable analysis, men had worse OS (HR: 1.38; 95% CI 1:15–1.64) and recurrence-free survival (HR: 1.40; 95% CI 1.18–1.67), compared to women. On the contrary, when researchers overlooked non-cancer causes of death, CRC outcomes appeared similar in both genders. Additionally, they did not find sex-related significant differences regarding the CSS^19^.

On the contrary, a cross-sectional study conducted in the UK revealed that males had slightly better 1-year survival than females but the 5-year survival appeared similar between both sexes^17^. Although we found consistently worse OS, CSS, and NCSS for male sex across all cancer stages, White et al. showed inconsistent results. They demonstrated that 1-year survival was similar in both genders diagnosed at stages I and II while females had a survival advantage in stages III and IV. Moreover, they claimed comparable 5-year survival for both males and females diagnosed with I, III, and IV stages, yet females had better survival for stage II^17^. However, these contradictory results may be owing to White et al. adjusting the data for age, without adjustment for demographic factors such as ethnicity and socioeconomic status which may be connected to the CRC detection and outcome including survival^20–23^. A recently published population-based study from the SEER database revealed that metastatic EOCRC had longer survival than metastatic late-onset CRC patients (p < 0.0001). In line with our findings, Ren et al. illustrated that females had superior survival rates than males among metastatic EOCRC (p < 0.001). However, they did not find a significant difference in the metastatic late-onset CRC (p = 0.57). They also concluded that sex-related differences in metastatic CRC survival correlate to patients’ age^4^.

Incidence and mortality rates are considered higher in men than in women^24^. Molecular and genetic factors, sex hormones, and lifestyle may be attributed to the favorable survival in females than males^25,26^. The increased vulnerability of males can be partially attributed to worse health choices such as smoking^27^ and heavier alcohol consumption rates compared to females^28^. Moreover, men are more inclined to consume a fatty diet and processed meat^29^. They also tend to develop visceral obesity^30^ which is considered a potential risk factor for CRC. A meta-analysis showed that CRC risk increases by 7% for each 2 km/m^2^ increase in the Body Mass Index (BMI) and increases by 4% for each 2 cm increase in waist circumference^31^, which is consistent with Bassett et al. findings^32^. The accumulation of all these risk factors in males might explain the worse NCSS observed in our study owing to the increased risk of non-cancer mortality due to worse health choices.

Li et al. also proposed that sex differences can be attributed to male-specific genes that are carried on the Y-chromosome and can be a determinant for CRC hallmarks and outcomes. They generated a murine CRC model engineered with an induced transgene encoding mutant KRAS oncogene (KRAS*) and conditional null alleles of *Apc* and *Trp53* tumor suppressors. They found higher metastasis rates and shorter survival in males compared to females. Furthermore, the molecular and transcriptomic analysis revealed that KRAS* mediated Signal Transducer and Activator of Transcription 4 (STAT4) transcription factor activation leading to the upregulation of one of the histone demethylases, Lysine Demethylase 5D (KDM5D) gene, encoded in the Y-chromosome. In turn, these transcriptomic changes repress genes regulating cell–cell junction integrity and CD8^+^ T Cell anti-tumor function. Interestingly, KDM5D deletion from cancer cells may decrease cancer invasiveness and enhance CD8^+^ T cell-killing activity; hence, it can be a promising therapeutic approach for CRC male patients expressing mutant KRAS^33^. Adding to the possible role of the immune system in EOCRC, a study by Ugai T et al. examined immune cell profiles in CRC patients. Comprehensive immunologic analyses following surgical resection in EOCRC patients were characterized by lower levels of tumor-infiltrating lymphocytes, intratumoral, periglandular, and peritumoral lymphocytic reaction. These findings underscore the importance of immune cell profile analysis based on age at diagnosis to better understand the unique pathogenesis of CRC in young adults^34^. Furthermore, some studies identified that sex is a considerable determinant of immunity^35^. For instance, estrogen has been identified as a regulator for the immune microenvironment of liver metastasis^36^; meanwhile, Schalper et found estradiol (E2) to increase programmed death ligand (PD-L1) expression in breast and endometrial cancer; hence, allowing cancer to escape immunosurveillance^37^. In this context, the IMMUNOREACT 5 trial investigated the difference in rectal mucosa immune microenvironment between both sexes. They observed that male patients have more mutations of *SYNE1* and *RYR2* oncogenes associated with lower expression of genes mediating T-cell activation. On the other hand, healthy female mucosa had more Th1 and cytotoxic T cells suggesting probably a better immune response against tumor cells^38^.

Lin et al. illustrated that high testosterone level was associated with decreased risk for CRC in men (RR 0.62; 95% CI 0.40–0.96). However, there is an inverse association between estradiol to testosterone ratio and CRC in postmenopausal women^39^. This could possibly explain the findings of Ren et al. concerning EOCRC having longer survival by eight months compared to LOCRC^4^, supporting that estrogen may have a protective effect in CRC. Nevertheless, researchers found that estrogen protects against microsatellite instability (MSI) and gene methylation in colon tumors. Therefore, females are less likely to develop MSI + colon cancer at a younger age during their reproductive period than males while being at higher risk of developing unstable tumors at older ages owing to the reduction of estrogen levels^40^. Still, benefits from hormonal replacement therapy, particularly in postmenopausal women, may be attenuated with breast cancer, cardiovascular diseases, and thromboembolism^41^. Estrogen receptor 1 (ESR1) is primarily expressed in breast cancer and promotes metastasis. Unlike ESR1, estrogen receptor 2 (ESR2) is expressed in CRC and is associated with tumor suppression. Liu et al.^42^ demonstrated that the WAP Four-Disulfide Core Domain 3 (WFDC3) gene regulates ESR2 which, in turn, represses Transforming Growth Factor Beta Receptor 1 (TGFBR1) and inhibits CRC metastasis^42^. Hence, targeting the ESR2 pathway can be a promising therapeutic approach^42^. However, further investigations to understand the underlying mechanism of this pathway and develop new therapeutic hits.

Previously reported evidence may justify our findings of worse survivals in male patients and across all subgroups, owing to behavioral, genetic, and immunological factors, as well as sex hormones. Our analysis confirmed that sex is an independent prognostic factor in EOCRC. Sex differences may comprise a fundamental difference between both sexes in terms of pathogenesis and response to treatment. So, we recommend further controlled clinical trials that assess responses to different interventions between males and females separately. Moreover, evaluating the existence of some biological biomarkers may direct treatment decisions and even predict survival. Overall, we believe understanding the epidemiological and molecular basis of sex differences in EOCRC will enable targeted and precision medicine; hence, reducing EOCRC’s emerging burden on communities. Current therapeutic guidelines recommend the use of aggressive treatment regimens for EOCRC patients^43,44^. A recent multi-center analysis showed that EOCRC patients benefit at least the same as, or even more than, older-onset CRC from CRC-directed treatment modalities^45^.

To our knowledge, the current research is the first to investigate sex differences in EOCRC survival with PSM analysis and sex-specific predictors of survival from US population-level data. Our analysis revealed that radiotherapy predicts better survival in both sexes. Other demographic and patient characteristics were almost comparable, with some exceptions. However, we think more research is needed to confirm the reproducibility of the results. there is also a need for a more comprehensive sex-specific prediction model of survival that determines survival according to the molecular and hormonal basis of the disease. Admittedly, our study has its limitations such as possible biases introduced by its retrospective nature. Our analysis did not take into consideration certain patients’ information such as detailed medical history and comorbidities, social status, and impactful health habits as these data were not available in the SEER database. Additionally, in the current study, we defined EOCRC as patients diagnosed between the ages of 20 and 49 which may be inconsistent with some literature that used the age of 15 as a lower cut-off. Furthermore, we used the SEER staging variable which is known for most of the patients in the database to limit missing data and maintain a large sample size in our study bypassing any possible inconsistencies between different American Joint Committee on Cancer (AJCC) staging editions for patients in the SEER database. Future studies should account for AJCC staging which is more clinically widely acceptable.

## Conclusions

To conclude, we found significant differences in baseline variables between females and males at the time of EOCRC diagnosis. Additionally, male sex was associated with worse OS, CSS, and NCSS in both the general cohort and the post-PSM dataset. Furthermore, this analysis found that the majority of prognostic factors impacting survival outcomes of males and females diagnosed with EOCRC are comparable except for some minor differences.

## Supplementary Information

Supplementary Tables.

## Data Availability

Data were retrieved and extracted from the publicly available SEER database. Access to the SEER database is available through https://seer.cancer.gov/. Individual patient data used in the analysis cannot be shared according to a signed “no release” agreement mandatory by the SEER database. However, researchers can access the original datasets upon completion of research data agreements and authorization steps conducted by the National cancer institute staff according to the SEER database policy (https://seer.cancer.gov/data/access.htm).

## Abbreviations

EOCRC: Early-onset colorectal cancer
CRC: Colorectal cancer
LOCRC: Later-onset colorectal cancer
SEER: Surveillance, epidemiology, and end results
OS: Overall survival
CSS: Cancer-specific survival
NCSS: Noncancer-specific survival
HR: Hazard ratio
CI: Confidence interval
PSM: Propensity score matching
STROBE: Strengthening the reporting of observational studies in epidemiology
BMI: Body mass index
MSI: Microsatellite instability
ESR1: Estrogen receptor 1
ESR2: Estrogen receptor 2
TGFBR1: Transforming growth factor beta receptor 1
WFDC3: WAP four-disulfide core domain 3
PD-L1: Programmed death-ligand 1
STAT4: Signal transducer and activator of transcription 4
KDM5D: Lysine demethylase 5D
ICD-0–3: International classification of diseases for oncology, third edition
WHO: World health organization
AJCC: American joint committee on cancer
