# Supplementary material for: Sex differences in survival outcomes of early-onset colorectal cancer
Source: Sci Rep. 2024 Sep 26;14:22041. doi: 10.1038/s41598-024-71999-8 (PMC11427454; doi:10.1038/s41598-024-71999-8)
Supplement: Supplementary file 1 — Supplementary Tables. [file 41598_2024_71999_MOESM1_ESM.docx]

**Supplementary table 1. Cox regression models of survival in the general cohort (unmatched) cohort.**

|  | **OS** | | | | **CSS** | | | | **NCSS** | | | |
| --- | --- | --- | --- | --- | --- | --- | --- | --- | --- | --- | --- | --- |
|  | **Univariable HR (95% CI)** | **p-value** | **Multivariable HR (95% CI)** | **p-value** | **Univariable HR (95% CI)** | **p-value** | **Multivariable HR (95% CI)** | **p-value** | **Univariable HR (95% CI)** | **p-value** | **Multivariable HR (95% CI)** | **p-value** |
| **Sex** |  |  |  |  |  |  |  |  |  |  |  |  |
| Female | Ref | - | Ref | - | Ref | - | Ref | - | Ref | - | Ref | - |
| Male | 1.19 (1.16-1.22) | <0.001 | 1.19 (1.16-1.22) | <0.001 | 1.15 (1.12-1.19) | <0.001 | 1.15 (1.11-1.18) | <0.001 | 1.52 (1.41-1.65) | <0.001 | 1.56 (1.44-1.69) | <0.001 |
| **Age** |  |  |  |  |  |  |  |  |  |  |  |  |
| 20-29 | Ref | - | Ref | - | Ref | - | Ref | - | Ref | - | Ref | - |
| 30-39 | 1.11 (1.04-1.19) | 0.002 | 1.02 (0.95-1.09) | 0.62 | 1.10 (1.03-1.18) | 0.008 | 0.97 (0.91-1.04) | 0.435 | 1.35 (1.05-1.74) | 0.018 | 1.58 (1.23-2.04) | <0.001 |
| 40-49 | 1.23 (1.16-1.31) | <0.001 | 1.16 (1.08-1.23) | <0.001 | 1.17 (1.10-1.25) | <0.001 | 1.06 (1.00-1.14) | 0.066 | 2.07 (1.64-2.62) | <0.001 | 2.47 (1.95-3.14) | <0.001 |
| **Race** |  |  |  |  |  |  |  |  |  |  |  |  |
| White | Ref | - | Ref | - | Ref | - | Ref | - | Ref | - | Ref | - |
| Black | 1.36 (1.31-1.40) | <0.001 | 1.28 (1.24-1.33) | <0.001 | 1.34 (1.29-1.39) | <0.001 | 1.28 (1.24-1.33) | <0.001 | 1.51 (1.37-1.67) | <0.001 | 1.27 (1.14-1.40) | <0.001 |
| Asian or Pacific Islander | 0.99 (0.95-1.04) | 0.824 | 1.05 (1.00-1.09) | 0.057 | 1.03 (0.99-1.08) | 0.172 | 1.07 (1.02-1.12) | 0.006 | 0.70 (0.60-0.82) | <0.001 | 0.81 (0.69-0.95) | 0.009 |
| American Indian/Alaska Native | 1.19 (1.05-1.33) | 0.005 | 0.90 (0.78-1.03) | 0.128 | 1.11 (0.98-1.27) | 0.103 | 0.86 (0.74-1.00) | 0.051 | 1.76 (1.31-2.35) | <0.001 | 1.43 (1.01-2.04) | 0.045 |
| Unspecified | 0.11 (0.07-0.16) | <0.001 | 0.20 (0.14-0.30) | <0.001 | 0.11 (0.07-0.16) | <0.001 | 0.24 (0.16-0.35) | <0.001 | 0.07 (0.02-0.29) | <0.001 | 0.08 (0.02-0.32) | <0.001 |
| **Marital Status** |  |  |  |  |  |  |  |  |  |  |  |  |
| Married | Ref | - | Ref | - | Ref | - | Ref | - | Ref | - | Ref | - |
| Single | 1.44 (1.40-1.49) | <0.001 | 1.31 (1.27-1.36) | <0.001 | 1.39 (1.35-1.44) | <0.001 | 1.25 (1.21-1.30) | <0.001 | 1.92 (1.76-2.10) | <0.001 | 1.92 (1.76-2.10) | <0.001 |
| Unmarried | 1.41 (1.36-1.47) | <0.001 | 1.29 (1.24-1.35) | <0.001 | 1.33 (1.27-1.39) | <0.001 | 1.21 (1.16-1.27) | <0.001 | 2.18 (1.95-2.44) | <0.001 | 2.08 (1.86-2.32) | <0.001 |
| Unspecified | 0.80 (0.75-0.85) | <0.001 | 0.94 (0.88-1.01) | 0.072 | 0.76 (0.71-0.81) | <0.001 | 0.93 (0.87-1.00) | 0.046 | 1.16 (0.97-1.38) | 0.108 | 1.08 (0.90-1.30) | 0.385 |
| **Income** |  |  |  |  |  |  |  |  |  |  |  |  |
| < $35k | Ref | - | Ref | - | Ref | - | Ref | - | Ref | - | Ref | - |
| $35k - $50k | 0.98 (0.87-1.11) | 0.767 | 0.94 (0.84-1.07) | 0.355 | 1.00 (0.88-1.14) | 0.957 | 0.97 (0.85-1.10) | 0.611 | 0.86 (0.63-1.17) | 0.349 | 0.82 (0.60-1.12) | 0.204 |
| $50k - $75k | 0.85 (0.76-0.95) | 0.004 | 0.89 (0.78-1.00) | 0.05 | 0.89 (0.79-1.01) | 0.062 | 0.92 (0.81-1.05) | 0.222 | 0.61 (0.46-0.83) | 0.001 | 0.66 (0.48-0.91) | 0.01 |
| >$75k | 0.72 (0.64-0.81) | <0.001 | 0.78 (0.69-0.88) | <0.001 | 0.76 (0.68-0.87) | <0.001 | 0.82 (0.71-0.93) | 0.003 | 0.47 (0.35-0.63) | <0.001 | 0.54 (0.39-0.74) | <0.001 |
| **Area of Residence** |  |  |  |  |  |  |  |  |  |  |  |  |
| Metropolitan | Ref | - | Ref | - | Ref | - | Ref | - | Ref | - | Ref | - |
| Nonmetropolitan | 1.16 (1.12-1.21) | <0.001 | 1.09 (1.04-1.15) | <0.001 | 1.13 (1.09-1.18) | <0.001 | 1.09 (1.03-1.15) | 0.002 | 1.42 (1.28-1.58) | <0.001 | 1.10 (0.96-1.26) | 0.168 |
| Unspecified | 1.05 (0.83-1.33) | 0.68 | 1.84 (1.40-2.41) | <0.001 | 0.95 (0.73-1.23) | 0.682 | 1.76 (1.30-2.38) | <0.001 | 1.87 (1.11-3.16) | 0.019 | 1.78 (0.94-3.36) | 0.075 |
| **Primary Site** |  |  |  |  |  |  |  |  |  |  |  |  |
| Right-sided colon | Ref | - | Ref | - | Ref | - | Ref | - | Ref | - | Ref | - |
| Transverse colon | 1.07 (1.00-1.14) | 0.053 | 1.04 (0.98-1.11) | 0.213 | 1.05 (0.98-1.13) | 0.131 | 1.03 (0.96-1.10) | 0.423 | 1.14 (0.95-1.36) | 0.154 | 1.14 (0.96-1.36) | 0.144 |
| left-sided colon | 0.97 (0.94-1.01) | 0.12 | 0.99 (0.95-1.03) | 0.511 | 0.99 (0.95-1.02) | 0.452 | 0.99 (0.95-1.03) | 0.596 | 0.88 (0.79-0.97) | 0.013 | 0.92 (0.82-1.02) | 0.105 |
| Rectum/Rectosigmoid junction | 0.92 (0.89-0.95) | <0.001 | 0.92 (0.89-0.96) | <0.001 | 0.93 (0.90-0.96) | <0.001 | 0.94 (0.90-0.98) | 0.004 | 0.84 (0.76-0.92) | <0.001 | 0.78 (0.69-0.88) | <0.001 |
| Unspecified | 2.26 (2.10-2.42) | <0.001 | 1.38 (1.28-1.49) | <0.001 | 2.33 (2.16-2.51) | <0.001 | 1.38 (1.28-1.49) | <0.001 | 1.71 (1.35-2.16) | <0.001 | 1.32 (1.04-1.68) | 0.025 |
| **Grade** |  |  |  |  |  |  |  |  |  |  |  |  |
| Grade I | Ref | - | Ref | - | Ref | - | Ref | - | Ref | - | Ref | - |
| Grade II | 2.02 (1.91-2.15) | <0.001 | 1.50 (1.41-1.59) | <0.001 | 2.25 (2.11-2.40) | <0.001 | 1.58 (1.48-1.69) | <0.001 | 1.14 (1.00-1.30) | 0.052 | 1.12 (0.97-1.28) | 0.115 |
| Grade III | 3.94 (3.70-4.19) | <0.001 | 2.36 (2.22-2.52) | <0.001 | 4.63 (4.32-4.96) | <0.001 | 2.58 (2.40-2.77) | <0.001 | 1.30 (1.11-1.53) | 0.001 | 1.23 (1.04-1.45) | 0.015 |
| Grade IV | 4.13 (3.75-4.55) | <0.001 | 2.60 (2.36-2.87) | <0.001 | 4.75 (4.29-5.26) | <0.001 | 2.80 (2.52-3.10) | <0.001 | 1.68 (1.22-2.30) | 0.001 | 1.54 (1.12-2.12) | 0.007 |
| Unspecified | 2.27 (2.13-2.42) | <0.001 | 1.47 (1.38-1.57) | <0.001 | 2.51 (2.33-2.69) | <0.001 | 1.53 (1.42-1.65) | <0.001 | 1.35 (1.16-1.57) | <0.001 | 1.21 (1.03-1.41) | 0.018 |
| **Histology** |  |  |  |  |  |  |  |  |  |  |  |  |
| Adenocarcinomas | Ref | - | Ref | - | Ref | - | Ref | - | Ref | - | Ref | - |
| Cystic/mucinous | 1.65 (1.59-1.72) | <0.001 | 1.20 (1.16-1.25) | <0.001 | 1.72 (1.65-1.79) | <0.001 | 1.22 (1.17-1.27) | <0.001 | 1.17 (1.04-1.33) | 0.012 | 1.09 (0.96-1.24) | 0.182 |
| Others | 1.74 (1.62-1.88) | <0.001 | 1.13 (1.04-1.22) | 0.003 | 1.71 (1.58-1.86) | <0.001 | 1.10 (1.01-1.20) | 0.024 | 1.96 (1.59-2.40) | <0.001 | 1.41 (1.13-1.75) | 0.002 |
| **Stage** |  |  |  |  |  |  |  |  |  |  |  |  |
| Localized | Ref | - | Ref | - | Ref | - | Ref | - | Ref | - | Ref | - |
| Regional | 2.86 (2.73-2.99) | <0.001 | 2.65 (2.52-2.79) | <0.001 | 4.15 (3.92-4.39) | <0.001 | 3.73 (3.51-3.96) | <0.001 | 1.05 (0.97-1.15) | 0.221 | 1.20 (1.08-1.32) | <0.001 |
| Distant | 16.59  (15.88-17.33) | <0.001 | 12.52  (11.90-13.17) | <0.001 | 26.10  (24.71-27.58) | <0.001 | 18.86  (17.74-20.06) | <0.001 | 1.78 (1.58-2.01) | <0.001 | 1.88 (1.64-2.16) | <0.001 |
| Unspecified | 3.20 (2.92-3.51) | <0.001 | 1.92 (1.74-2.11) | <0.001 | 4.33 (3.89-4.82) | <0.001 | 2.56 (2.29-2.86) | <0.001 | 1.64 (1.36-1.99) | <0.001 | 1.21 (0.98-1.50) | 0.077 |
| **Radiotherapy** |  |  |  |  |  |  |  |  |  |  |  |  |
| No/Unknown | Ref | - | Ref | - | Ref | - | Ref | - | Ref | - | Ref | - |
| Yes | 1.18 (1.15-1.22) | <0.001 | 1.09 (1.05-1.13) | <0.001 | 1.20 (1.16-1.24) | <0.001 | 1.09 (1.04-1.13) | <0.001 | 1.01 (0.92-1.11) | 0.818 | 1.30 (1.15-1.48) | <0.001 |
| **Chemotherapy** |  |  |  |  |  |  |  |  |  |  |  |  |
| No/Unknown | Ref | - | Ref | - | Ref | - | Ref | - | Ref | - | Ref | - |
| Yes | 2.45 (2.38-2.52) | <0.001 | 0.92 (0.89-0.96) | <0.001 | 2.92 (2.83-3.02) | <0.001 | 0.96 (0.93-1.00) | 0.065 | 0.85 (0.79-0.92) | <0.001 | 0.65 (0.59-0.72) | <0.001 |
| **Surgery** |  |  |  |  |  |  |  |  |  |  |  |  |
| No/Unknown | Ref | - | Ref | - | Ref | - | Ref | - | Ref | - | Ref | - |
| Yes | 0.23 (0.23-0.24) | <0.001 | 0.40 (0.39-0.41) | <0.001 | 0.22 (0.21-0.23) | <0.001 | 0.39 (0.38-0.40) | <0.001 | 0.52 (0.46-0.58) | <0.001 | 0.62 (0.54-0.71) | <0.001 |

**OS, Overall Survival; CSS, Cancer-Specific Survival; NCSS, Noncancer-Specific Survival; HR, Hazard Ratio; CI, Confidence Interval; Ref, Reference.**

**Supplementary table 2. Baseline characteristics of the** **post-propensity score matching (PSM) cohort.**

|  | **Female (N=26103)** | **Male (N=26103)** | **p-value** |
| --- | --- | --- | --- |
| **Age** |  |  | 0.734_1_ |
| 20-29 | 1479.0 (5.7%) | 1457.0 (5.6%) |  |
| 30-39 | 5587.0 (21.4%) | 5654.0 (21.7%) |  |
| 40-49 | 19037.0 (72.9%) | 18992.0 (72.8%) |  |
| **Race** |  |  | 0.849_1_ |
| White | 19182.0 (73.5%) | 19294.0 (73.9%) |  |
| Black | 3775.0 (14.5%) | 3730.0 (14.3%) |  |
| Asian or Pacific Islander | 2595.0 (9.9%) | 2543.0 (9.7%) |  |
| American Indian/Alaska Native | 276.0 (1.1%) | 265.0 (1.0%) |  |
| Unspecified | 275.0 (1.1%) | 271.0 (1.0%) |  |
| **Marital Status** |  |  | 0.735_1_ |
| Married | 15087.0 (57.8%) | 15201.0 (58.2%) |  |
| Single | 6759.0 (25.9%) | 6694.0 (25.6%) |  |
| Unmarried | 2686.0 (10.3%) | 2633.0 (10.1%) |  |
| Unspecified | 1571.0 (6.0%) | 1575.0 (6.0%) |  |
| **Income** |  |  | 0.554_1_ |
| < $35k | 301.0 (1.2%) | 283.0 (1.1%) |  |
| $35k - $50k | 2499.0 (9.6%) | 2500.0 (9.6%) |  |
| $50k - $75k | 13069.0 (50.1%) | 12946.0 (49.6%) |  |
| >$75k | 10234.0 (39.2%) | 10374.0 (39.7%) |  |
| **Area of Residence** |  |  | 0.399_1_ |
| Metropolitan | 23059.0 (88.3%) | 23150.0 (88.7%) |  |
| Nonmetropolitan | 2977.0 (11.4%) | 2882.0 (11.0%) |  |
| Unspecified | 67.0 (0.3%) | 71.0 (0.3%) |  |
| **Primary Site** |  |  | 0.476_1_ |
| Right-sided colon | 6591.0 (25.2%) | 6504.0 (24.9%) |  |
| Transverse colon | 1225.0 (4.7%) | 1181.0 (4.5%) |  |
| left-sided colon | 7767.0 (29.8%) | 7756.0 (29.7%) |  |
| Rectum/Rectosigmoid junction | 9928.0 (38.0%) | 10098.0 (38.7%) |  |
| Unspecified | 592.0 (2.3%) | 564.0 (2.2%) |  |
| **Grade** |  |  | 0.546_1_ |
| Grade I | 2970.0 (11.4%) | 2887.0 (11.1%) |  |
| Grade II | 14665.0 (56.2%) | 14820.0 (56.8%) |  |
| Grade III | 3822.0 (14.6%) | 3832.0 (14.7%) |  |
| Grade IV | 535.0 (2.0%) | 508.0 (1.9%) |  |
| Unspecified | 4111.0 (15.7%) | 4056.0 (15.5%) |  |
| **Histology** |  |  | 0.807_1_ |
| Adenocarcinomas | 22896.0 (87.7%) | 22942.0 (87.9%) |  |
| Cystic/mucinous | 2633.0 (10.1%) | 2602.0 (10.0%) |  |
| Others | 574.0 (2.2%) | 559.0 (2.1%) |  |
| **Stage** |  |  | 0.403_1_ |
| Localized | 8967.0 (34.4%) | 8961.0 (34.3%) |  |
| Regional | 9848.0 (37.7%) | 9989.0 (38.3%) |  |
| Distant | 6594.0 (25.3%) | 6500.0 (24.9%) |  |
| Unspecified | 694.0 (2.7%) | 653.0 (2.5%) |  |
| **Radiotherapy** |  |  | 0.835_1_ |
| No/Unknown | 20943.0 (80.2%) | 20924.0 (80.2%) |  |
| Yes | 5160.0 (19.8%) | 5179.0 (19.8%) |  |
| **Chemotherapy** |  |  | 0.192_1_ |
| No/Unknown | 11512.0 (44.1%) | 11364.0 (43.5%) |  |
| Yes | 14591.0 (55.9%) | 14739.0 (56.5%) |  |
| **Surgery** |  |  | 0.071_1_ |
| No/Unknown | 3312.0 (12.7%) | 3176.0 (12.2%) |  |
| Yes | 22791.0 (87.3%) | 22927.0 (87.8%) |  |

**Supplementary table 3. Cox regression models of survival in the post-propensity score matching (PSM) cohort.**

|  | **OS** | | | | **CSS** | | | | **NCSS** | | | |
| --- | --- | --- | --- | --- | --- | --- | --- | --- | --- | --- | --- | --- |
|  | **Univariable HR (95% CI)** | **P-VALUE** | **Multivariable HR (95% CI)** | **p-value** | **Univariable HR (95% CI)** | **p-value** | **Multivariable HR (95% CI)** | **p-value** | **Univariable HR (95% CI)** | **P-VALUE** | **Multivariable HR (95% CI)** | **p-value** |
| **Sex** |  |  |  |  |  |  |  |  |  |  |  |  |
| Female | Ref | - | Ref | - | Ref | - | Ref | - | Ref | - | Ref | - |
| Male | 1.12 (1.09-1.16) | < 0.001 | 1.18 (1.15-1.21) | < 0.001 | 1.08 (1.05-1.11) | <0.001 | 1.14 (1.10-1.17) | <0.001 | 1.51 (1.39-1.64) | < 0.001 | 1.58 (1.45-1.71) | < 0.001 |
| **Age** |  |  |  |  |  |  |  |  |  |  |  |  |
| 20-29 | Ref | - | Ref | - | Ref | - | Ref | - | Ref | - | Ref | - |
| 30-39 | 1.14 (1.06-1.22) | 0.001 | 1.02 (0.95-1.10) | 0.57 | 1.13 (1.04-1.21) | 0.002 | 0.98 (0.90-1.05) | 0.519 | 1.31 (1.00-1.71) | 0.048 | 1.58 (1.21-2.07) | 0.001 |
| 40-49 | 1.24 (1.16-1.32) | < 0.001 | 1.17 (1.10-1.26) | < 0.001 | 1.17 (1.09-1.26) | <0.001 | 1.08 (1.00-1.16) | 0.041 | 2.03 (1.58-2.61) | < 0.001 | 2.51 (1.95-3.24) | < 0.001 |
| **Race** |  |  |  |  |  |  |  |  |  |  |  |  |
| White | Ref | - | Ref | - | Ref | - | Ref | - | Ref | - | Ref | - |
| Black | 1.36 (1.31-1.41) | < 0.001 | 1.28 (1.23-1.33) | < 0.001 | 1.34 (1.29-1.39) | <0.001 | 1.28 (1.23-1.34) | <0.001 | 1.54 (1.39-1.71) | < 0.001 | 1.24 (1.11-1.38) | < 0.001 |
| Asian or Pacific Islander | 1.00 (0.96-1.05) | 0.892 | 1.06 (1.01-1.12) | 0.013 | 1.04 (0.99-1.09) | 0.153 | 1.08 (1.03-1.14) | 0.002 | 0.74 (0.63-0.87) | < 0.001 | 0.86 (0.73-1.02) | 0.081 |
| American Indian/Alaska Native | 1.18 (1.03-1.34) | 0.014 | 0.88 (0.76-1.02) | 0.101 | 1.09 (0.94-1.26) | 0.255 | 0.84 (0.72-0.99) | 0.043 | 1.88 (1.38-2.56) | < 0.001 | 1.44 (0.98-2.11) | 0.063 |
| Unspecified | 0.10 (0.06-0.15) | < 0.001 | 0.18 (0.12-0.28) | < 0.001 | 0.10 (0.06-0.16) | <0.001 | 0.21 (0.14-0.34) | <0.001 | 0.08 (0.02-0.33) | < 0.001 | 0.09 (0.02-0.36) | 0.001 |
| **Marital Status** |  |  |  |  |  |  |  |  |  |  |  |  |
| Married | Ref | - | Ref | - | Ref | - | Ref | - | Ref | - | Ref | - |
| Single | 1.41 (1.36-1.45) | < 0.001 | 1.32 (1.28-1.37) | < 0.001 | 1.35 (1.30-1.40) | <0.001 | 1.26 (1.21-1.30) | <0.001 | 1.92 (1.75-2.11) | < 0.001 | 1.98 (1.80-2.18) | < 0.001 |
| Unmarried | 1.47 (1.41-1.54) | < 0.001 | 1.29 (1.24-1.35) | < 0.001 | 1.38 (1.32-1.45) | <0.001 | 1.21 (1.16-1.27) | <0.001 | 2.29 (2.03-2.57) | < 0.001 | 2.10 (1.86-2.36) | < 0.001 |
| Unspecified | 0.78 (0.73-0.84) | < 0.001 | 0.95 (0.89-1.02) | 0.198 | 0.74 (0.69-0.80) | <0.001 | 0.94 (0.88-1.02) | 0.144 | 1.15 (0.96-1.39) | 0.135 | 1.09 (0.90-1.32) | 0.387 |
| **Income** |  |  |  |  |  |  |  |  |  |  |  |  |
| < $35k | Ref | - | Ref | - | Ref | - | Ref | - | Ref | - | Ref | - |
| $35k - $50k | 1.01 (0.89-1.16) | 0.825 | 0.94 (0.82-1.07) | 0.331 | 1.07 (0.93-1.23) | 0.355 | 0.99 (0.86-1.14) | 0.886 | 0.76 (0.56-1.04) | 0.087 | 0.69 (0.50-0.95) | 0.024 |
| $50k - $75k | 0.86 (0.76-0.98) | 0.02 | 0.89 (0.78-1.01) | 0.073 | 0.93 (0.81-1.07) | 0.311 | 0.95 (0.82-1.10) | 0.515 | 0.54 (0.40-0.73) | < 0.001 | 0.56 (0.40-0.77) | < 0.001 |
| >$75k | 0.73 (0.64-0.82) | < 0.001 | 0.77 (0.68-0.89) | < 0.001 | 0.80 (0.69-0.91) | 0.001 | 0.84 (0.73-0.98) | 0.025 | 0.41 (0.30-0.55) | < 0.001 | 0.45 (0.32-0.62) | < 0.001 |
| **Area of Residence** |  |  |  |  |  |  |  |  |  |  |  |  |
| Metropolitan | Ref | - | Ref | - | Ref | - | Ref | - | Ref | - | Ref | - |
| Nonmetropolitan | 1.18 (1.13-1.23) | < 0.001 | 1.11 (1.06-1.17) | < 0.001 | 1.15 (1.10-1.21) | <0.001 | 1.12 (1.06-1.18) | <0.001 | 1.41 (1.26-1.58) | < 0.001 | 1.07 (0.92-1.24) | 0.372 |
| Unspecified | 1.03 (0.78-1.34) | 0.852 | 1.82 (1.33-2.47) | < 0.001 | 0.85 (0.62-1.17) | 0.323 | 1.61 (1.13-2.30) | 0.008 | 2.34 (1.38-3.96) | 0.002 | 2.17 (1.13-4.17) | 0.02 |
| **Primary Site** |  |  |  |  |  |  |  |  |  |  |  |  |
| Right-sided colon | Ref | - | Ref | - | Ref | - | Ref | - | Ref | - | Ref | - |
| Transverse colon | 1.05 (0.98-1.13) | 0.127 | 1.03 (0.96-1.11) | 0.367 | 1.05 (0.97-1.13) | 0.22 | 1.02 (0.95-1.10) | 0.569 | 1.11 (0.91-1.34) | 0.3 | 1.12 (0.92-1.35) | 0.252 |
| left-sided colon | 0.96 (0.93-1.00) | 0.055 | 0.98 (0.94-1.02) | 0.359 | 0.98 (0.94-1.02) | 0.241 | 0.98 (0.94-1.03) | 0.474 | 0.88 (0.78-0.98) | 0.017 | 0.90 (0.81-1.01) | 0.074 |
| Rectum/Rectosigmoid junction | 0.86 (0.83-0.90) | < 0.001 | 0.92 (0.88-0.96) | < 0.001 | 0.87 (0.84-0.91) | <0.001 | 0.93 (0.89-0.98) | 0.004 | 0.81 (0.73-0.90) | < 0.001 | 0.77 (0.68-0.87) | < 0.001 |
| Unspecified | 2.17 (2.00-2.34) | < 0.001 | 1.33 (1.23-1.45) | < 0.001 | 2.26 (2.08-2.45) | <0.001 | 1.34 (1.23-1.46) | <0.001 | 1.50 (1.15-1.96) | 0.002 | 1.23 (0.93-1.61) | 0.143 |
| **Grade** |  |  |  |  |  |  |  |  |  |  |  |  |
| Grade I | Ref | - | Ref | - | Ref | - | Ref | - | Ref | - | Ref | - |
| Grade II | 2.01 (1.89-2.13) | < 0.001 | 1.51 (1.42-1.61) | < 0.001 | 2.24 (2.09-2.40) | <0.001 | 1.60 (1.49-1.72) | <0.001 | 1.12 (0.98-1.29) | 0.103 | 1.12 (0.97-1.29) | 0.116 |
| Grade III | 3.96 (3.70-4.23) | < 0.001 | 2.37 (2.21-2.53) | < 0.001 | 4.69 (4.35-5.04) | <0.001 | 2.59 (2.40-2.79) | <0.001 | 1.26 (1.06-1.49) | 0.009 | 1.23 (1.03-1.47) | 0.021 |
| Grade IV | 4.23 (3.82-4.68) | < 0.001 | 2.62 (2.36-2.90) | < 0.001 | 4.93 (4.43-5.49) | <0.001 | 2.84 (2.54-3.17) | <0.001 | 1.50 (1.06-2.12) | 0.023 | 1.41 (0.99-2.00) | 0.056 |
| Unspecified | 2.18 (2.04-2.34) | < 0.001 | 1.48 (1.38-1.59) | < 0.001 | 2.41 (2.23-2.60) | <0.001 | 1.55 (1.43-1.68) | <0.001 | 1.31 (1.12-1.54) | 0.001 | 1.19 (1.01-1.41) | 0.033 |
| **Histology** |  |  |  |  |  |  |  |  |  |  |  |  |
| Adenocarcinomas | Ref | - | Ref | - | Ref | - | Ref | - | Ref | - | Ref | - |
| Cystic/mucinous | 1.68 (1.61-1.75) | < 0.001 | 1.18 (1.13-1.23) | < 0.001 | 1.75 (1.68-1.83) | <0.001 | 1.20 (1.15-1.25) | <0.001 | 1.14 (1.00-1.31) | 0.054 | 1.09 (0.94-1.25) | 0.254 |
| Others | 1.79 (1.65-1.95) | < 0.001 | 1.16 (1.07-1.26) | 0.001 | 1.77 (1.63-1.94) | <0.001 | 1.14 (1.04-1.25) | 0.005 | 1.92 (1.54-2.40) | < 0.001 | 1.39 (1.10-1.76) | 0.006 |
| **Stage** |  |  |  |  |  |  |  |  |  |  |  |  |
| Localized | Ref | - | Ref | - | Ref | - | Ref | - | Ref | - | Ref | - |
| Regional | 2.89 (2.75-3.03) | < 0.001 | 2.71 (2.57-2.86) | < 0.001 | 4.28 (4.02-4.55) | <0.001 | 3.87 (3.62-4.13) | <0.001 | 1.04 (0.95-1.13) | 0.452 | 1.23 (1.10-1.37) | < 0.001 |
| Distant | 16.91 (16.14-17.72) | < 0.001 | 12.93 (12.25-13.65) | < 0.001 | 27.15 (25.60-28.80) | <0.001 | 19.80 (18.54-21.16) | <0.001 | 1.80 (1.59-2.04) | < 0.001 | 1.95 (1.69-2.26) | < 0.001 |
| Unspecified | 3.06 (2.76-3.39) | < 0.001 | 1.92 (1.73-2.14) | < 0.001 | 4.22 (3.75-4.76) | <0.001 | 2.62 (2.31-2.96) | <0.001 | 1.53 (1.23-1.89) | < 0.001 | 1.15 (0.91-1.45) | 0.25 |
| **Radiotherapy** |  |  |  |  |  |  |  |  |  |  |  |  |
| No/Unknown | Ref | - | Ref | - | Ref | - | Ref | - | Ref | - | Ref | - |
| Yes | 1.15 (1.12-1.19) | < 0.001 | 1.08 (1.03-1.12) | 0.001 | 1.18 (1.14-1.22) | <0.001 | 1.07 (1.02-1.12) | 0.004 | 0.98 (0.88-1.09) | 0.72 | 1.32 (1.14-1.51) | < 0.001 |
| **Chemotherapy** |  |  |  |  |  |  |  |  |  |  |  |  |
| No/Unknown | Ref | - | Ref | - | Ref | - | Ref | - | Ref | - | Ref | - |
| Yes | 2.53 (2.45-2.61) | < 0.001 | 0.93 (0.89-0.96) | < 0.001 | 3.06 (2.96-3.17) | <0.001 | 0.98 (0.94-1.02) | 0.271 | 0.83 (0.76-0.90) | < 0.001 | 0.62 (0.55-0.69) | < 0.001 |
| **Surgery** |  |  |  |  |  |  |  |  |  |  |  |  |
| No/Unknown | Ref | - | Ref | - | Ref | - | Ref | - | Ref | - | Ref | - |
| Yes | 0.23 (0.23-0.24) | < 0.001 | 0.40 (0.39-0.42) | < 0.001 | 0.22 (0.21-0.23) | <0.001 | 0.39 (0.38-0.41) | <0.001 | 0.49 (0.43-0.56) | < 0.001 | 0.56 (0.48-0.65) | < 0.001 |

**OS, Overall Survival; CSS, Cancer-Specific Survival; NCSS, Noncancer-Specific Survival; HR, Hazard Ratio; CI, Confidence Interval; Ref, Reference.**

**Supplementary table 4. Cox regression models of survival of males (vs females) in different subgroups.**

|  | **OS** | | | | **CSS** | | | | **NCSS** | | | |
| --- | --- | --- | --- | --- | --- | --- | --- | --- | --- | --- | --- | --- |
|  | **Univariable HR (95% CI)** | **p-value** | **Multivariable HR (95% CI)** | **p-value** | **Univariable HR (95% CI)** | **p-value** | **Multivariable HR (95% CI)** | **p-value** | **Univariable HR (95% CI)** | **P-value** | **Multivariable HR (95% CI)** | **p-value** |
| **Age** |  |  |  |  |  |  |  |  |  |  |  |  |
| 20-29 | 1.27 (1.12-1.43) | <0.001 | 1.17 (1-03-1.33) | 0.013 | 1.3 (1.14-1.47) | <0.001 | 1.19 (1.05-1.35) | 0.009 | 0.94 (0.59-1.5) | 0.802 | 0.96 (0.6-1.54) | 0.863 |
| 30-39 | 1.19 (1.12-1.26) | <0.001 | 1.22 (1.15-1.30) | <0.001 | 1.19 (1.12-1.27) | <0.001 | 1.22 (1.15-1.3) | <0.001 | 1.19 (0.98-1.45) | 0.075 | 1.22 (1-1.49) | 0.049 |
| 40-49 | 1.18 (1.15-1.22) | <0.001 | 1.18 (1.15-1.22) | <0.001 | 1.13 (1.1-1.17) | <0.001 | 1.12 (1.09-1.16) | <0.001 | 1.62 (1.48-1.76) | <0.001 | 1.67 (1.52-1.82) | <0.001 |
| **Race** |  |  |  |  |  |  |  |  |  |  |  |  |
| White | 1.21 (1.17-1.25) | <0.001 | 1.21 (1.17-1.25) | <0.001 | 1.17 (1.13-1.21) | <0.001 | 1.16 (1.12-1.2) | <0.001 | 1.59 (1.45-1.75) | <0.001 | 1.58 (1.44-1.74) | <0.001 |
| Black | 1.25 (1.17-1.33) | <0.001 | 1.17 (1.10-1.25) | <0.001 | 1.22 (1.14-1.3) | <0.001 | 1.13 (1.05-1.2) | 0.003 | 1.5 (1.26-1.79) | <0.001 | 1.5 (1.26-1.8) | <0.001 |
| Asian or Pacific Islander | 1.05 (0.96-1.14) | 0.276 | 1.1 (1-1.2) | 0.04 | 1.02 (0.93-1.12) | 0.65 | 1.07 (0.98-1.1) | 0.136 | 1.44 (1.06-1.95) | 0.021 | 1.42 (1.04-1.94) | 0.027 |
| American Indian/Alaska Native | 1.29 (1.02-1.63) | 0.037 | 1.11 (0.86-1.42) | 0.43 | 1.26 (0.97-1.63) | 0.081 | 1.05 (0.8-1.37) | 0.714 | 1.43 (0.8-2.57) | 0.226 | 1.47 (0.79-2.72) | 0.219 |
| **Marital Status** |  |  |  |  |  |  |  |  |  |  |  |  |
| Married | 1.12 (1.08-1.16) | <0.001 | 1.16 (1.12-1.2) | <0.001 | 1.08 (1.04-1.13) | <0.001 | 1.13 (1.08-1.17) | <0.001 | 1.49 (1.33-1.68) | <0.001 | 1.49 (1.33-1.68) | <0.001 |
| Single | 1.25 (1.19-1.31) | <0.001 | 1.19 (1.14-1.25) | <0.001 | 1.22 (1.16-1.28) | <0.001 | 1.15 (1.09-1.21) | <0.001 | 1.52 (1.31-1.73) | <0.001 | 1.54 (1.34-1.77) | <0.001 |
| Unmarried | 1.33 (1.23-1.43) | <0.001 | 1.27 (1.18-1.37) | <0.001 | 1.25 (1.16-1.36) | <0.001 | 1.19 (1.09-1.29) | <0.001 | 1.82 (1.5-2.21) | <0.001 | 1.8 (1.48-2.19) | <0.001 |
| **Income** |  |  |  |  |  |  |  |  |  |  |  |  |
| < $35k | 1.09 (0.87-1.36) | 0.476 | 0.97 (0.76-1.23) | 0.779 | 1.13 (0.88-1.45) | 0.328 | 1.01 (0.78-1.32) | 0.92 | 0.87 (0.49-1.55) | 0.631 | 0.77 (0.41-1.43) | 0.409 |
| $35k - $50k | 1.25 (1.16-1.35) | <0.001 | 1.18 (1.09-1.28) | <0.001 | 1.21 (1.11-1.32) | <0.001 | 1.12 (1.03-1.23) | 0.007 | 1.56 (1.25-1.94) | <0.001 | 1.55 (1.24-1.93) | <0.001 |
| $50k - $75k | 1.23 (1.18-1.27) | <0.001 | 1.25 (1.20-1.30) | <0.001 | 1.18 (1.14-1.23) | <0.001 | 1.2 (1.15-1.25) | <0.001 | 1.64 (1.46-1.83) | <0.001 | 1.65 (1.47-1.84) | <0.001 |
| >$75k | 1.14 (1.09-1.19) | <0.001 | 1.12 (1.07-1.17) | <0.001 | 1.11 (1.06-1.16) | <0.001 | 1.08 (1.03-1.13) | <0.001 | 1.45 (1.27-1.66) | <0.001 | 1.49 (1.31-1.71) | <0.001 |
| **Area of Residence** |  |  |  |  |  |  |  |  |  |  |  |  |
| Metropolitan | 1.19 (1.16-1.22) | <0.001 | 1.2 (1.16-1.23) | <0.001 | 1.15 (1.12-1.19) | <0.001 | 1.15 (1.12-1.19) | <0.001 | 1.53 (1.4-1.67) | <0.001 | 1.57 (1.44-1.71) | <0.001 |
| Nonmetropolitan | 1.19 (1.11-1.28) | <0.001 | 1.14 (1.06-1.23) | <0.001 | 1.15 (1.06-1.24) | <0.001 | 1.09 (1.01-1.18) | 0.033 | 1.54 (1.26-1.88) | <0.001 | 1.51 (1.23-1.86) | <0.001 |
| **Primary Site** |  |  |  |  |  |  |  |  |  |  |  |  |
| Right-sided colon | 1.07 (1.02-1.13) | 0.008 | 1.17 (1.11-1.24) | <0.001 | 1.04 (0.98-1.1) | 0.207 | 1.13 (1.07-1.2) | <0.001 | 1.39 (1.19-1.63) | <0.001 | 1.47 (1.26-1.72) | <0.001 |
| Transverse colon | 0.99 (0.88-1.12) | 0.89 | 1.03 (0.91--1.16) | 0.67 | 0.95 (0.83-1.07) | 0.388 | 0.97 (0.86-1.11) | 0.682 | 1.35 (0.97-1.87) | 0.073 | 1.39 (0.99-1.94) | 0.054 |
| left-sided colon | 1.15 (1.09-1.21) | <0.001 | 1.13 (1.08-1.19) | <0.001 | 1.1 (1.04-1.16) | <0.001 | 1.08 (1.03-1.14) | 0.003 | 1.64 (1.42-1.91) | <0.001 | 1.62 (1.39-1.88) | <0.001 |
| Rectum/Rectosigmoid junction | 1.36 (1.3-1.42) | <0.001 | 1.25 (1.2-1.31) | <0.001 | 1.34 (1.28-1.4) | <0.001 | 1.21 (1.16-1.27) | <0.001 | 1.54 (1.35-1.75) | <0.001 | 1.61 (1.41-1.84 | <0.001 |
| **Grade** |  |  |  |  |  |  |  |  |  |  |  |  |
| Grade I | 1.38 (1.23-1.54) | <0.001 | 1.37 (1.22-1.54) | <0.001 | 1.35 (1.19-1.53) | <0.001 | 1.33 (1.17-1.51) | <0.001 | 1.5 (1.17-1.93) | 0.001 | 1.54 (1.2-1.99) | 0.001 |
| Grade II | 1.17 (1.13-1.21) | <0.001 | 1.17 (1.13-1.21) | <0.001 | 1.12 (1.08-1.16) | <0.001 | 1.11 (1.07-1.16) | <0.001 | 1.65 (1.48-1.83) | <0.001 | 1.69 (1.52-1.89) | <0.001 |
| Grade III | 1.05 (1-1.11) | 0.06 | 1.11 (1.05-1.18) | <0.001 | 1.05 (0.99-1.11) | 0.122 | 1.11 (1.04-1.17) | 0.001 | 1.17 (0.95-1.44) | 0.143 | 1.21 (0.97-1.49) | 0.087 |
| Grade IV | 1.17 (1-1.36) | 0.053 | 1.2 (1.02-1.41) | 0.029 | 1.14 (0.97-1.34) | 0.12 | 1.18 (1-2.39) | 0.056 | 1.63 (0.9-2.95) | 0.108 | 1.49 (0.81-2.74) | 0.197 |
| **Histology** |  |  |  |  |  |  |  |  |  |  |  |  |
| Adenocarcinomas | 1.2 (1.17-1.24) | <0.001 | 1.18 (1.14-1.21) | <0.001 | 1.16 (1.13-1.2) | <0.001 | 1.13 (1.09-1.16) | <0.001 | 1.58 (1.45-1.73) | <0.001 | 1.62 (1.48-1.76) | <0.001 |
| Cystic/mucinous | 1.02 (0.95-1.09) | 0.54 | 1.15 (1.07-1.23) | <0.001 | 1.01 (0.94-1.08) | 0.872 | 1.13 (1.05-1.22) | 0.001 | 1.21 (0.95-1.53) | 0.124 | 1.22 (0.96-1.56) | 0.106 |
| **Stage** |  |  |  |  |  |  |  |  |  |  |  |  |
| Localized | 1.59 (1.46-1.72) | <0.001 | 1.56 (1.44-1.69) | <0.001 | 1.52 (1.37-1.69) | <0.001 | 1.43 (1.29-1.59) | <0.001 | 1.68 (1.48-1.91) | <0.001 | 1.76 (1.55-2) | <0.001 |
| Regional | 1.24 (1.18-1.3) | <0.001 | 1.2 (1.14-1.25) | <0.001 | 1.2 (1.14-1.26) | <0.001 | 1.15 (1.1-1.21) | <0.001 | 1.46 (1.29-1.66) | <0.001 | 1.48 (1.31-1.68) | <0.001 |
| Distant | 1.15 (1.11-1.19) | <0.001 | 1.11 (1.07-1.15) | <0.001 | 1.14 (1.1-1.18) | <0.001 | 1.1 (1.06-1.14) | <0.001 | 1.38 (1.13-1.67) | 0.001 | 1.38 (1.13-1.68) | 0.002 |
| **Radiotherapy** |  |  |  |  |  |  |  |  |  |  |  |  |
| Yes | 1.19 (1.12-1.25) | <0.001 | 1.2 (1.13-1.26) | <0.001 | 1.15 (1.09-1.22) | <0.001 | 1.16 (1.09-1.23) | <0.001 | 1.56 (1.31-1.87) | <0.001 | 1.65 (1.37-1.97) | <0.001 |
| **Chemotherapy** |  |  |  |  |  |  |  |  |  |  |  |  |
| Yes | 1.1 (1.07-1.13) | <0.001 | 1.14 (1.1-1.18) | <0.001 | 1.07 (1.04-1.11) | <0.001 | 1.11 (1.08-1.15) | <0.001 | 1.5 (1.34-1.69) | <0.001 | 1.54 (1.37-1.73) | <0.001 |
| **Surgery** |  |  |  |  |  |  |  |  |  |  |  |  |
| Yes | 1.14 (1.11-1.18) | <0.001 | 1.19 (1.15-1.22) | <0.001 | 1.09 (1.05-1.12) | <0.001 | 1.13 (1.09-1.17) | <0.001 | 1.56 (1.44-1.7) | <0.001 | 1.62 (1.42-1.77) | <0.001 |

**OS, Overall Survival; CSS, Cancer-Specific Survival; NCSS, Noncancer-Specific Survival; HR, Hazard Ratio; CI, Confidence Interval; Ref, Reference.**
